# The Efficacy of Gait Training Using a Body Weight Support Treadmill and Visual Biofeedback in Patients with Subacute Stroke: A Randomized Controlled Trial

**DOI:** 10.1155/2018/3812602

**Published:** 2018-04-05

**Authors:** Mariusz Drużbicki, Grzegorz Przysada, Agnieszka Guzik, Agnieszka Brzozowska-Magoń, Krzysztof Kołodziej, Andzelina Wolan-Nieroda, Joanna Majewska, Andrzej Kwolek

**Affiliations:** ^1^Institute of Physiotherapy, University of Rzeszów, Warszawska 26a, Rzeszów, Poland; ^2^Clinical Rehabilitation Ward of Province Hospital No. 2 in Rzeszów, Lwowska 60, Rzeszów, Poland; ^3^Centre for Innovative Research in Medical and Natural Sciences, University of Rzeszów, Warzywna 1a, 35-310 Rzeszów, Poland

## Abstract

**Background:**

This study was designed to determine whether or not gait training based on the use of treadmill with visual biofeedback and body weight support (BWS) would produce better effects in patients with subacute stroke compared to BWS treadmill training with no visual biofeedback.

**Materials and Methods:**

30 patients with subacute stroke were randomly assigned to do body weight supported treadmill training with visual biofeedback (BB group) or BWS treadmill training without visual biofeedback. Their gait was assessed with a 3D system (spatiotemporal gait parameters and symmetry index) and by means of 2-minute walk test (2 MWT), 10-metre walk test (10 MWT), and Timed Up & Go test. Subjects in both groups participated in 15 treadmill training sessions (30 minutes each).

**Results:**

The participants from both groups achieved a statistically significant improvement in spatiotemporal gait parameters, walking speed, endurance, and mobility. The average change in the BB group after the end of the programme did not differ significantly compared to the change in the control group. The change in the symmetry index value of stance phase in the BB group was 0.03 (0.02) and in the control group was 0.02 (0.02). The difference was not statistically significant (*p* = 0.902). The statistically significantly higher improvement in the BB group was found in the range of walking speed (*p* = 0.003) and endurance (*p* = 0.012), but the difference between groups was of low clinical significance.

**Conclusions:**

The findings do not confirm that BWS treadmill training with the function of visual biofeedback leads to significantly greater improvement in gait compared to BWS treadmill training with no visual biofeedback at an early stage after stroke. This study was registered at ClinicalTrials.gov, ID: ACTRN12616001283460.

## 1. Introduction

Despite considerable progress in treatment and prevention methods, stroke continues to be one of the main causes of death and permanent disability [1]. It is assumed that two in three stroke survivors will experience functional limitations, such as gait impairment, and will need rehabilitative care [2]. Gait abnormalities due to hemiplegia result in reduced gait speed, distance, and efficiency, leading to significantly limited functional performance. Walking ability in patients after stroke is characterised by asymmetry of parameters describing gait patterns. Gait asymmetry is observed in over 30% of individuals with stroke. The problem is mainly reflected by asymmetry of step length, asymmetry of stance phase with longer duration of stance on nonparetic leg, asymmetry of swing phase duration, double and single support time, and asymmetry in the range of motion in the joints of the lower limbs [3, 4].

Recovery of independent and safe gait pattern, as similar to normal walking competency as possible, is therefore one of the main purposes of poststroke rehabilitation [5–7].

Motor reeducation and gait reeducation must be optimally matched to the stage of stroke recovery in terms of intensity, frequency, and duration of exercise, and the tasks must gradually be more difficult and more demanding [8]. In restoration of gait function, basic physical therapy may be supplemented with treatments based on electromechanical devices such as treadmills, body weight support systems, and robot assisted gait trainers [9].

It has been established that treadmill gait training both with and without BWS enables improvement in walking speed and distance, and the effects are maintained after the intervention ends [10, 11]. A systematic review by Mehrholz et al. shows that, compared to over-ground walking training, treadmill gait training with or without BWS does not increase the chances for regaining the ability to walk independently, yet ambulatory patients may, as a result of treadmill training, have significantly improved walking speed and endurance and the benefits persist after the end of the training [12, 13].

To reduce the asymmetry of the parameters, gait reeducation programmes also apply split-belt treadmill training. It has been shown that this method can successfully be used to improve step length symmetry in patients with chronic stroke [14].

Methods used in motor reeducation following injury of the central nervous system include biofeedback techniques providing patients with real-time biological information which in the person's current functional condition would otherwise be unknown [15, 16]. Biofeedback systems most often perform measurement of biomedical variables and communicate these to the user (patient), directly or indirectly, the relevant variable (strength, movement, myoelectric potential, and temperature) being transformed into a visual, acoustic, or any other feedback signal which is easy to interpret [17]. Applied in gait reeducation, visual or acoustic feedback provides the patient with information on the current temporospatial, kinetic, and kinematic gait parameters. Research has shown that treadmill training with acoustic biofeedback divided into three separate time series, corresponding to three pacing frequencies, leads to improved gait coordination in hemiplegic patients with chronic stroke [18]. It has also been reported that treadmill training with visual biofeedback administered for 15–30 min per day, for two weeks, gives better results in improving gait cycle length, duration of gait phases, and swing phase speed compared to exercise on a treadmill alone, in poststroke hemiplegic patients at a chronic stage [19].

The use of visual biofeedback and treadmill was studied by Lewek et al. They reported a significant improvement in temporospatial gait symmetry in a group of subjects with chronic stroke, after 6-week training. A drawback of the study was the fact that the group using treadmill and visual biofeedback was compared to a group participating in a conventional gait training programme [20].

 Brasileiro et al. also conducted a study in a group of patients with chronic stroke, yet the randomly selected groups received BWS treadmill gait training alone, or accompanied with functions of either visual (step length visualization) or auditory biofeedback. The authors reported that visual and auditory biofeedback combined with BWS treadmill training did not produce significant effects in terms of improved temporospatial parameters and gait symmetry in patients with chronic stroke. A previous study by the present authors focused on subjects with chronic stroke [21]. The applied treadmill training with velocity progression enabled significant improvement in temporospatial and kinematic gait parameters and a decrease in asymmetry in both groups. Based on these findings, it was impossible to determine whether a greater effect was produced by the visual biofeedback or the treadmill training alone [22].

Stanton et al. in a systematic review demonstrated moderate effects of biofeedback in improved motor control of the paretic lower limb, mainly with regard to improved standing, walking, and balance [23]. Majority of the studies reviewed by Stanton et al. investigated patients with subacute to chronic stroke. Effects of the applied therapies were assessed taking mainly into account walking speed, step length, functional performance, and balance. None of the studies discussed in the review assessed symmetry of gait pattern and its changes after treadmill training. To our knowledge, there are no up-to-date studies assessing the effects of visual biofeedback in the improvement of gait in patients with subacute stroke in terms of temporospatial and kinematic gait parameters. Before the study, we formulated a hypothesis that, by introducing treadmill training combined with visual biofeedback, providing accurate real-time information on the length of step performed with paretic and nonparetic leg, it will be possible to achieve greater improvement in gait symmetry in patients at an early stage after stroke, compared to the basic gait training. It was also anticipated that gait reeducation at an early stage after stroke, supplemented with additional external information, would lead to long-lasting improvement which will be sustained after the end of the programme.

The study was designed to determine whether or not gait training based on the use of treadmill with visual biofeedback and BWS would produce better effects in patients with subacute stroke compared to BWS treadmill training with no visual biofeedback.

## 2. Materials and Methods

### 2.1. Study Design

The data presented in this article were obtained in a two-armed randomized controlled trial, assessing gait in patients with subacute stroke. The study was approved by the Local Bioethics Commission of the Medical Faculty, University of Rzeszów (17/02/2016), and was registered with the Australian New Zealand Clinical Trials Registry (ACTRN12616001283460). Experimental conditions conformed to the Declaration of Helsinki and all participants gave their informed written consent to participate in the study.

Single blind trial design was applied with randomized assignment of participants to one of the two parallel groups comprising a total of 30 patients at an early stage after stroke, receiving treatment in a rehabilitation clinic. Simple randomization was used, whereby the patients were allocated either to the group exercising on a treadmill with body weight support and visual feedback (BB group) or to the group exercising on a BWS treadmill without visual feedback (the control group). Each participant completed 15 treadmill training sessions in a course of three consecutive weeks.

### 2.2. Setting

The study was carried out in the Clinical Rehabilitation Ward with Early Neurological Rehabilitation Unit, at the Province Hospital No. 2 in Rzeszów, Poland.

### 2.3. Participants

The study was conducted among patients admitted into the clinic in the period from January to December 2016. 30 patients with subacute stroke were included in the study. They were selected to participate in the programme by a physician with at least 10-year clinical experience in neurological rehabilitation. Inclusion criteria were as follows: (a) first ischemic stroke, (b) up to 30 days from stroke onset, (c) Brunnström stages of stroke recovery from 2 to 3, and (d) ability to walk unassisted. Exclusion criteria were as follows: (a) second or another stroke, (b) reported incident of severe heart failure and uncontrolled arterial hypertension, (c) cognitive disorders impairing the understanding of and ability to follow instructions (Mini-Mental State Examination score below 24), (d) visual field disturbances caused by a stroke or other visual disturbances impairing normal vision, (e) orthopaedic disorders significantly affecting the subjects' gait, and (f) untreated deep vein thrombosis.

### 2.4. Blinding Procedures

Each selected patient was randomly allocated either to the group exercising on a BWS treadmill and receiving visual feedback (BB group) or to the group exercising on a BWS treadmill without visual feedback (the control group). The participants were randomized using a table of random numbers (SAS software) in the BB group (*n* = 15) or the control group (*n* = 15). The person handling the randomization was also responsible for the list of patients divided into the groups and for informing the physiotherapist in charge of the training which patient was to do body weight supported gait training with visual feedback and which one was to do BWS treadmill training without visual feedback. The person administering the treadmill exercises was not involved in examining or assessing the patients. Initial and final gait assessments were conducted by a physiotherapist who did not know the subjects' assignment to the group and was not involved in the training. The list of patients divided into groups was kept by the researcher in charge of randomization. The list was decoded after the last of the qualified patients was assessed during the final exam. Before the start of the programme, the team of researchers was instructed not to share any information regarding the assessments and the course of treadmill training. 

## 3. Interventions

### 3.1. Intervention Procedures

The patients randomly allocated to the BB group completed 15 training sessions during three consecutive weeks (from Monday to Friday). A single exercise continued for 30 minutes per training session. The used treadmill, that is, Gait Trainer 3 (Biodex Medical Systems, USA), was equipped with a body weight support system (Biodex Medical Systems). Walking speed and step length were defined individually for each patient. The minimum treadmill walking speed was 0.34 m/s (the lowest velocity necessary for starting the visual biofeedback function).

During the first treadmill training session, gait speed was defined individually for each participant, based on the gait speed determined with 3D gait assessment during the initial examination. If the gait speed score was lower than 0.34 m/s (2 subjects in the BB group), during the first training session, the speed was gradually increased to 0.34 m/s (the minimum velocity necessary for starting the visual biofeedback function). Step length, the same for the right and the left leg, was determined during the first or the second training session. The initial step length, automatically selected by the treadmill software, was decreased to the value matching the subject's ability to walk with the same step length of the paretic and the nonparetic leg at the defined velocity. During consecutive sessions, step length and gait speed were increased by 5 to 10% of the value from the previous session. The new value was kept if the patient was able to maintain step length symmetry; otherwise, the training was conducted using the same step length and velocity as in the previous session.

**Figure 1:**
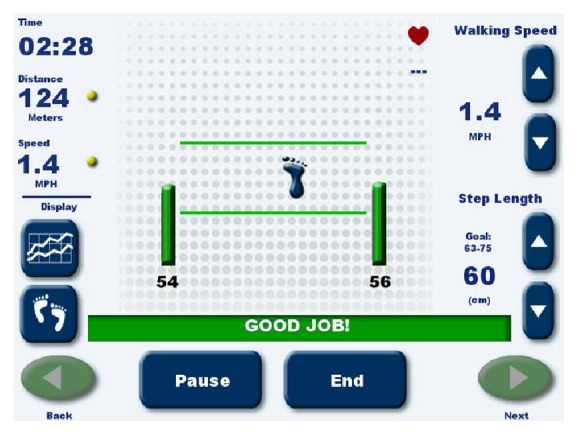
Treadmill Gait Trainer 3, Footfall Screen (Biodex Medical Systems).

The treadmill used in the programme is equipped with force sensors and software for marking the place on the treadmill where the load is exerted during stance phase by the right and the left leg. Information reflecting the actual location of feet was presented to the patients in the form of graphic visualization (foot contours) on a screen. During the training, the patient was expected to match the length of step performed with the right and the left leg to the task displayed on the screen, in the form of two parallel lines between which the feet were to be positioned. The distance between the lines corresponded to the step length defined by the physiotherapist. During exercise, the patient was asked to walk with such speed and step length as to make sure that the graphic representations of the right and the left foot are located within the area between the lines (Figure 1). Additional feedback on correct or incorrect performance was communicated with a text message displayed on the monitor.

The initial body weight support was set at 30%. As treatment progressed, the body weight support was gradually decreased and the velocity was gradually increased. The training parameters were based on recommendation from previous literature [24, 25].

Partial body weight support was used during all the training sessions. During treadmill training, the patients were wearing their own shoes and were allowed to use orthopaedic aids (AFOs).

During the exercise, the patients were asked to refrain from holding onto the handrails of the treadmill. The physiotherapist in charge did not provide manual support; the patients walked without help (hand support only if required).

The participants randomly allocated to the control group completed 15 training sessions during three consecutive weeks. A single training session was 30 minutes long. The same treadmill was used along with the same system of partial body weight support, yet the biofeedback function was not applied. The screen of the control panel informed the patients about the current timing of the exercise and the distance covered so far. The rate of body weight support was defined at the level of 30%, like in the group exercising with biofeedback. The patients walked on the treadmill without help and were allowed to use AFOs.

During the first treadmill training session, gait speed was defined based on the 3D gait assessment score determined during the initial examination. During consecutive sessions, gait speed was increased by 5 to 10% provided that this increase did not adversely impact the gait mechanism and did not cause excessive fatigue. The participants from the control group during treadmill training received current information on the walking speed, duration of the session, and the distance covered. The controls were not informed about step length (the information is available only through the biofeedback function).

All the treadmill sessions were supervised by a physician. On admission to the clinic, the patients were subjected to ECG examination; measurement of blood pressure and pulse was performed before each training session, every 5–7 minutes, and after each training session. The physician decided on starting or interrupting the training. Pulse measuring sensors built into the rails were not used during the treadmill training. The participants were asked to report excessive fatigue or a need to rest. Patients could rest for 2-3 minutes if their heart rate exceeds the submaximal level, that is, 70% (HR submax = 0.75 × (220 − age)) or if they experienced fatigue.

Treadmill walk training was stopped when systolic blood pressure was 200 mmHg and diastolic blood pressure was 110 mmHg. There were short breaks during the training. No undesirable medical events caused by treadmill training occurred during the entire programme.

### 3.2. Conventional Rehabilitation

During their stay in the clinic, the patients from both groups received a rehabilitation programme, including individual physical therapy and occupational therapy. The physiotherapy programme comprised exercises designed to improve balance and stability in low positions, during sitting and standing, as well as gait training, including standing up and walking up the stairs. Individual physiotherapy also included hand therapy. Each participant of the programme took part in daily gait exercise performed in groups. The daily duration of physical therapy administered to each patient, excluding the treadmill training, totaled 120 minutes. No gait training aids were used in addition to the treadmill. Physical therapy was conducted in the clinic every day, from Monday to Saturday. On Saturday, physical therapy sessions were 45 minutes long. The exercises were administered by the clinic's physiotherapists with experience in working with patients with stroke. All the physiotherapists working with the participants of the programme had been trained in the use of PNF and NDT Bobath in hemiplegia. Occupational therapy was conducted every day and focused on recovery of basic activities of daily living.

### 3.3. Outcome Measurements

#### 3.3.1. Outcome Measures

Primary and secondary outcome measures were assessed at baseline and after 15 sessions of the intervention.

#### 3.3.2. Primary Outcomes: Gait Analysis

Primary outcomes were the spatiotemporal gait parameters. We evaluated step length (SL) that measured the average distance (in meters) between two successive placements of the same foot [26], the stance time (ST) that measured the duration of the stance phase (% stride), the swing time (SW) that measured the duration of the swing phase (% stride), gait speed (m/s), and the cadence (step/min) that measured the number of steps taken in a given period of time, which was then converted into the number of steps taken per minute [27]. 3D gait analysis was performed with six infrared cameras (BTS SMART-DX 700, BTS Bioengineering, Milano, Italy) and two force platforms (AMTI, USA). In accordance with Davis Marker Placement protocol, passive markers, reflecting IR radiation, were positioned on the sacrum, pelvis (anterior posterior iliac spine), femur (greater trochanter, lateral epicondyle, and lower 1/3 of the shank), fibula (lateral condyle, lateral malleolus, and lower 1/3 of the shank), and foot (heel metatarsal and head) [28]. 3D analysis took into account data obtained during six complete trips (complete cycle of the right and the left leg) at a distance of 8 metres. The analyses disregarded the trips with marker loss or lack of recording of ground reaction force. 3D analyses were performed with BTS Analyzer software (BTS Bioengineering, Milano, Italy). During the trials, the participants walked with a self-selected comfortable pace. They were allowed to use their own orthopaedic aids (AFOs, canes, and crutches) and during the exercise they were secured by the physiotherapist.

#### 3.3.3. Primary Outcomes: Gait Symmetry

In order to assess symmetry of gait parameters, the symmetry index (SI) [4] was calculated:

(1) $$\begin{array}{c} SI=\frac{V_{paretic}{\;\;-\;\;V}_{nonparetic}}{0.5\left(V_{paretic}+V_{nonparetic}\right)}, \end{array}$$

where *V*_paretic_ is a gait variable related to the paretic leg and *V*_nonparetic_ is a corresponding variable for the nonparetic leg. The value of SI represents the degree of asymmetry. SI value equal to zero reflects full symmetry. SI was calculated for SW phase (SI SW), ST phase (SI ST), and SL phase (SI SL).

#### 3.3.4. Secondary Outcomes: Gait Velocity

A secondary outcome was the gait velocity assessed by mean velocity (m/s), which measured the rate of change of position, recorded in metres per second [29]. The 10-metre walk test (10 MWT) was used to assess walking speed. High interrater and intrarater reliabilities have been established for timed walking tests, including the 10 MWT [30]. The subjects were to cover a defined distance of 10 metres twice, walking at a comfortable speed, using their own orthopaedic aids (AFOs, canes, or crutches). The duration was measured with a stopwatch [31, 32].

#### 3.3.5. Secondary Outcomes: Endurance

Endurance was assessed with the 2-minute walk test. The test was originally used in the assessment of patients with cardiovascular disorders. Later, it was adopted as a tool assessing the performance of senior patients and individuals with neurological conditions [33]. The trials were carried out in a hallway, free of any obstructions, where a distance of 25 metres was marked. The participant was asked to walk, at a comfortable pace, for two minutes between the defined points. The result of the test is given as a distance covered in metres. The participants were allowed to use their own orthopaedic aids (AFOs, canes, or crutches) [34, 35].

#### 3.3.6. Secondary Outcomes: Mobility

The Timed Up & Go (TUG) test was used to evaluate functional mobility [36]. TUG has been shown to be valid, reliable, and sensitive to changes, being recommended for measuring basic mobility skills in stroke subjects who are able to walk [37]. The subject was asked to stand up without help from a chair with armrests, walk a distance of 3 metres, turn round at a designated point, return to the chair, and sit down without help. The task was to be performed as quickly as possible. Two trials were performed and the mean score was calculated.

### 3.4. Statistical Methods

We undertook an a priori power calculation to determine sample size based on primary outcome measures and gait speed (3D analysis). This measure was chosen because it provides valuable insight into quality of gait and gait velocity is the most widely reported measure and is believed to be a good indicator of overall gait performance [38]. The minimal clinically important difference for gait speed in patients across the 20- to 60-day period after stroke was estimated previously at 0,16 m/s [39] and adopted here. To indicate difference between the groups and assuming the alpha level of 0.05 and power of 80%, it was calculated to allocate at least 14 subjects to each group.

Both parametric and nonparametric tests were applied in the analysis of the variables. The selection of a parametric test depended on the fulfilment of its basic assumptions, that is, distributions of the examined variables conforming with normal distribution, which was verified with Shapiro-Wilk *W*-test. Descriptive statistics, calculated for all numerical variables, included the mean, median, and standard deviation. Assessment of intragroup variability in the two populations was performed with Student's *t*-test for independent variables, or alternatively with nonparametric Wilcoxon signed-rank test. Differences in the average values of a numerical characteristic in the two populations were assessed with Student's *t*-test for independent variables, or alternatively with nonparametric Mann–Whitney *U* test. Statistical significance was assumed at *p* < 0.05. All calculations and statistical analyses were performed using STATISTICA ver. 10.0 (StatSoft, Poland).

## 4. Results

### 4.1. Flow of Included and Patients' Characteristics

**Figure 2:**
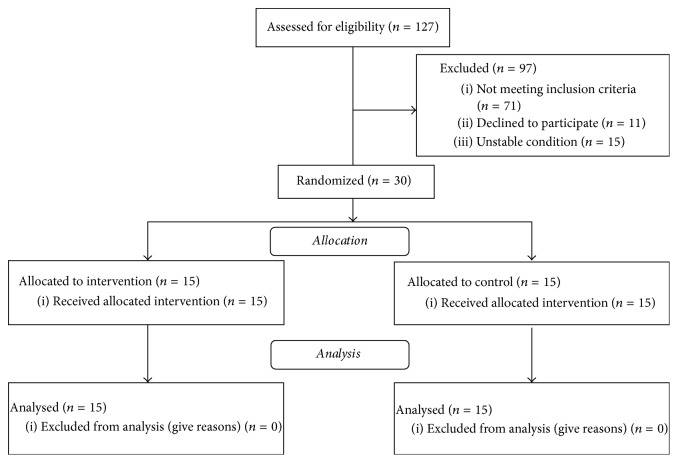
Study flow diagram.

127 patients were examined successively, as they were admitted to the Clinical Rehabilitation Ward. Inclusion criteria were met by 30 of them. Out of the 97 patients who were not qualified for the programme, 71 failed to meet the inclusion criteria, 15 were in an unstable condition, and 11 refused to participate (Figure 2). All the qualified patients took part in 15 training sessions and completed the programme. No undesirable medical events occurred during the programme. All the subjects completed the final examination.

Demographic and clinical characteristics are shown in Table 1. No statistically significant differences were found between the groups.

### 4.2. Outcome Measures

#### 4.2.1. Spatiotemporal Variables

The mean changes (difference between the mean score in the final exam and the mean value in the baseline exam) in the temporospatial gait parameters in the BB group and in the controls are shown in Table 2.

The stance phase on the nonparetic limb in the BB group on average increased by 7.53 (95% CI: 5.35–9.70); the controls were also found with longer duration of stance phase on the nonparetic limb, with the mean value of the increase amounting to 4.84 (95% CI: 2.69–6.99). The mean values of the change in the two groups did not differ significantly (*p* = 0.067). Similarly, no statistically significant differences between the BB group and the controls were observed in the duration of the swing phase on the paretic and nonparetic limb (SW paretic *p* = 0.089; SW nonparetic *p* = 0.074).

The change in the mean number of steps in the BB group amounted to 13.47 (95% CI: 9.17–17.76), compared to the mean change in the controls at the level of 6.48 (95% CI: 4.00–8.96). The difference between the groups was statistically significant (*p* = 0.007). The change in paretic and nonparetic step length was greater by 0.02 m in the control group. The difference between the groups was not statistically significant.

#### 4.2.2. Symmetry Index

The mean changes in the symmetry index of stance phase, swing phase, and step length are shown in Table 2. The mean change in stance phase symmetry index in the BB group was 0.03 (95% CI: 0.01–0.04), and in the controls the mean change in SI was 0.02 (95% CI: 0.01–0.03). The difference was statistically insignificant. Likewise, the mean changes in swing phase symmetry index did not differ between the two groups. The mean change in step length symmetry index in the BB group amounted to 0.08 (95% CI: 0.05–0.11), and in the controls the mean change in SI was 0.05 (95% CI: 0.03–0.06). The difference between the groups was statistically significant (*p* = 0.044).

### 4.3. Secondary Outcomes

The mean changes in gait speed (10 MWT [m/s]), walking distance (2 MWT [m]), and mobility (TUG [s]) in the BB group versus in the control group are presented in Table 3. In the BB group, the mean change was 0.07 m/s greater than in the controls (*p* = 0.003). The mean change in 2 MWT score in the BB group amounted to 25.73 m (95% CI: 19.03–32.44), compared to 14.0 m in the control group (95% CI: 7.44–20.56). The difference between the groups was statistically significant (*p* = 0.012). Assessment of mobility with TUG test showed a greater change in the control group. The difference was not statistically significant.

## 5. Discussion

Gait after stroke is characterised by a number of deficits, such as asymmetry of spatiotemporal parameters and decreased speed and endurance. Gait reeducation after stroke with the use of treadmill and BWS systems leads to an increase in gait speed and walking distance. The increase in gait speed results mainly from increased step length and cadence. On the other hand, no improvement in the symmetry of spatiotemporal parameters has been shown [40].

Patterson et al. in a retrospective study assessed changes in spatiotemporal gait asymmetry in 86 patients with stroke, who started rehabilitation in a period ranging from 19 to 30 days after stroke. Their findings did not show a statistically significant decrease in spatiotemporal parameters or a change in the direction of asymmetry. The authors emphasized that therapists were informed about the results of baseline gait assessment. They argued that the lack of improvement in symmetry was due to the fact that gait training at an early stage mainly aims at recovery of independent, functional, and safe walking skills rather than specifically at improving gait symmetry [41].

As it was mentioned in the Introduction, there are few reports describing studies which were designed to assess effects of therapy focusing on improvement of gait symmetry in patients at an early stage after stroke. The purpose of the present study was to determine whether or not the use of visual biofeedback in gait training at an early stage after stroke is an effective approach and if it facilitates improvement in walking ability, including spatiotemporal gait parameters.

The above study focused on subjects with chronic stroke, that is, individuals with persistent gait patterns, largely adapted to functioning in their own environment. Therefore, the subsequent study was intended to investigate the effectiveness of additional targeted therapy with the use of BWS treadmill training accompanied with visual biofeedback, to be introduced at an early stage after stroke and aimed at gait symmetry improvement. It was hypothesized that gait training at an early stage after stroke with the use of BWS treadmill and visual biofeedback will enable the subjects to restore more correct, symmetrical gait pattern. The present findings, however, do not support this hypothesis. After the end of the programme, the patients in the BB group and the controls changed the gait pattern. Both groups were found with significantly decreased stance phase on the nonparetic limb as well as longer paretic and nonparetic step length. Stance phase on the paretic limb in the BB group was slightly reduced. As a result, stance phase symmetry index was slightly improved in both groups. Likewise, both groups presented improved symmetry of swing phase duration, yet a statistically significant increase in swing phase was found only in the case of the nonparetic leg. Step length was significantly increased in the two groups, in both the paretic and the nonparetic leg. After the end of the programme, improved step length symmetry was found only in the BB group. Along with the improved spatiotemporal parameters, mainly related to the nonparetic leg, as well as enhanced symmetry of gait phases and step length, the patients in both groups presented significantly increased cadence and gait speed as well as endurance assessed with 2-minute walk test. Improved gait speed and endurance observed in both groups following the treadmill training are consistent with findings of many other studies [12]. Analysis of these results suggests that greater velocity may have been associated with the increased cadence and the increased step length. The greater gait speed and enhanced step length consequently led to longer stance phase on the nonparetic leg. Yet, the anticipated increase in the duration of stance phase on the paretic leg was not observed.

The changes in the relevant gait parameters, shown in each of the groups after the programme, do not provide evidence for a conclusion that visual biofeedback beneficially affects changes in gait patterns. Therefore, the mean changes in the spatiotemporal parameters and symmetry indexes in both groups were subjected to differential analysis. A statistically significant difference was only shown in the step length symmetry index, yet a greater mean change (increase in paretic and nonparetic step length) was found in the control group.

At the end of the BWS treadmill training, the subjects in both groups achieved a significant improvement in gait speed. The improvement in each of the groups exceeded the minimum, clinically meaningful difference defined by Tilson et al. for subjects between 20 and 60 days after stroke (0.16 m/s) [39]. Although the difference between the mean values of change was statistically significant, the fact that the value in the BB group was greater by 0.07 m/s is of little clinical importance [42]. The patients in the BB group also achieved greater improvement in endurance, assessed with 2 MWT, compared to the controls. The difference between the groups, however, was lower than the minimal detectable change defined by Rossier and Wade [35]. Therefore, the change may be of little clinical importance.

At the end of the therapy programme with the use of BWST with or without visual biofeedback, all the patients presented positive changes, mainly in the increased gait speed, endurance, and mobility. The quality of gait, reflected by the change in temporospatial parameters and their symmetry, was also improved, yet this was mainly a result of increased activity of the nonparetic leg. The changes were found in both groups, and no statistically significant differences between them were identified.

In view of the above, the study does not support the initial hypothesis anticipating that visual biofeedback accompanying BWS treadmill training will enable greater improvement in walking abilities at an early stage after stroke.

The study presents a few limitations, to be considered in further research. Firstly, the duration of treadmill training for only three weeks may have been too short for the subjects to acquire the desired gait pattern. What is more, it can be assumed that, at an early stage after stroke, a patient striving to regain the ability to walk unaided, during exercise performed independently, will focus more on attainment of the functional goals than on the quality of gait. The programme did not include monitoring of the amount of the patients' own exercise focusing on walking abilities and performed in addition to the therapy. It should be considered whether gait training oriented towards improvement of symmetry should not start after the patient regains independence in basic self-care activities. Further studies should be designed to supplement visual biofeedback assisted gait training with balance exercises supported with kinetic biofeedback informing on the loading of the paretic limb [43]. Importantly, future studies should also compare effects of using advanced techniques with visual biofeedback function to other cheaper methods providing additional visual or acoustic feedback during gait training.

Although our study has not presented evidence that the method of visual biofeedback facilitates improvement in gait function at an early stage after stroke, we believe that the use of the technique may be beneficial. Given the possible long-term effects [44], elimination of gait asymmetry should be a goal of gait reeducation at each stage of rehabilitation, including the early stage after stroke.

*Conclusions*. The findings do not confirm that BWS treadmill training with the function of visual biofeedback leads to significantly greater improvement in gait compared to BWS treadmill training with no visual biofeedback at an early stage after stroke.

## Figures and Tables

**Table 1:** Demographic data and baseline clinical characteristics.

Parameters	BB group	Control group
Age [years], mean (SD)	62.2 (10.2)	61.8 (11.1)
Sex [women/men]	5/10	7/8
Paretic limb [right/left]	8/7	8/7
Time from stroke, days, mean [range]	9 [6–23]	8 [5–19]

	Mean (SD)	Mean (SD)

ST nonparetic [%]	75.89 (5.43)	75.67 (5.60)
ST paretic [%]	64.09 (6.66)	65.72 (8.02)
*SI ST*	0.54 (0.02)	0.54 (0.02)
SW nonparetic [%]	24.45 (5.71)	24.33 (5.60)
SW paretic [%]	35.57 (7.04)	34.28 (8.02)
*SI SW*	0.60 (0.05)	0.59 (0.05)
Cadence [steps/min]	63.60 (9.91)	63.40 (14.77)
Gait speed [m/s]	0.38 (0.11)	0,39 (0.06)
SL nonparetic [m]	0.27 (0.12)	0.28 (0.08)
SL paretic [m]	0.23 (0.08)	0.21 (0.07)
*SI SL*	0.62 (0.06)	0.62 (0.04)

10 MWT [m/s]	0.36 (0.07)	0.37 (0.07)
2 MWT [m]	43.47 (7.47)	43.00 (4.77)
TUG test [s]	22.06 (5.03)	22.60 (2.77)

2 MWT: 2-minute walk test; 10 MWT: 10-metre walk test; SL: step length; SI: symmetry index; SW: swing phase; ST: stance phase; *p value*: result of the statistical test; BB: biofeedback group; $\overset{-}{x}$: mean; SD: standard deviation.

**Table 2:** Mean (standard deviation, 95% CI) change in the relevant parameters in the BB group and in the control group and the result of the statistical test.

Parameters	BB group		Control group		*p value*
	Mean (SD)	(95% CI)	Mean (SD)	(95% CI)	
ST nonparetic [%]	7.53 (3.93)	(5,35–9,70)	4.84 (3.88)	(2.69–6.99)	*0.067*
ST paretic [%]	6.11 (3.09)	(4.40–7,82)	4.52 (4.06)	(2.27–6.77)	*0.106*
*SI ST*	0.03 (0.02)	(0.01–0.04)	0.02 (0.02)	(0.01–0.03)	*0.902*
SW nonparetic [%]	7.23 (3.90)	(5.08–9.39)	4.84 (3.88)	(2.69–6.99)	*0.074*
SW paretic [%]	6.41 (3.24)	(4.61–8.20)	4.52 (4.06)	(2.27–6.77)	*0.089*
*SI SW*	0.06 (0.04)	(0.04–0.08)	0.04 (0.03)	(0.02–0.06)	*0.162*
Cadence [steps/min]	13.47 (7.76)	(9.17–17.76)	6.48 (4.47)	(4.00–8.96)	*0.007*
Gait speed [m/s]	0.38 (0.11)	(0,33–0,44)	0,39 (0.06)	(0,36–0,43)	*0,787*
SL nonparetic [m]	0.08 (0.08)	(0.03–0.12)	0.10 (0.07)	(0.06–0.14)	*0.126*
SL paretic [m]	0.10 (0.07)	(0.06–0.14)	0.12 (0.09)	(0.07–0.18)	*0.342*
*SI SL*	0.08 (0.06)	(0.05–0.11)	0.05 (0.03)	(0.03–0.06)	*0.044*

SL: step length; SI: symmetry index; SW: swing phase; ST: stance phase; *x*: mean; SD: standard deviation; *p value*: result of the statistical test; BB: biofeedback group.

**Table 3:** Mean, standard deviation, 95% CI, and result of the statistical test assessing changes in 10 MWT, 2 MWT, and TUG scores in the BB group and in the control group.

Parameters	BB group		Control group		*p value*
	Mean (SD)	(95% CI)	Mean (SD)	(95% CI)	
10 MWT [m/s]	0.27 (0.08)	0,14–0.25	0.2 (0.03)	0.07–0.17	*0.003*
2 MWT [m]	25.73 (12.11)	19.03–32.44	14.00 (11.85)	7.44–20.56	*0.012*
TUG test [s]	−6.70 (3.83)	4.58–8.82	−7.02 (3.03)	5.35–8.70	*0.799*

2 MWT: 2-minute walk test; 10 MWT: 10-metre walk test; *p value*: result of the statistical test; BB: biofeedback group; $\overset{-}{x}$: mean; SD: standard deviation.
